# Treatment in the home setting with intermittent pneumatic compression for a woman with chronic leg ulcers: a case report

**DOI:** 10.1186/s12912-017-0250-2

**Published:** 2017-09-21

**Authors:** Katrina Young, Harrison Ng Chok, Lesley Wilkes

**Affiliations:** 1 Nepean Hospital, Nepean Blue Mountains Local Health District, Derby Street, Kingswood, NSW 2747 Australia; 2 Western Sydney University/Nepean Blue Mountains Local Health District, Sydney, Australia; 3 Centre for Nursing Research and Practice Development, Nepean Hospital, First Floor - Court Building - Nepean Hospital, PO Box 63, Penrith, NSW 2751 Australia; 4 School of Nursing and Midwifery, Western Sydney University/Nepean Blue Mountains Local Health District, Sydney, Australia

**Keywords:** Intermittent Pneumatic Compression (IPC), Chronic Leg Ulcer (CLU), Venous Leg Ulcer (VLU), Chronic Venous Insufficiency (CVI), Lymphoedema, Case report, Home setting

## Abstract

**Background:**

Intermittent Pneumatic Compression (IPC) is shown to improve the healing rate of Venous Leg Ulcers (VLU) in the hospital setting. The current Australian “Gold Standard” treatment according to the Australian and New Zealand Wound Management Associations’ (AWMA) Prevention & Management of Venous Leg Ulcer guidelines is compression, generally in the form of bandaging then progressing to hosiery once wounds are healed to prevent recurrence. This is recommended in conjunction with other standards of wound management including; nutrition, exercise, client education and addressing underlying pathophysiology and psychosocial factors. Compression bandaging is predominantly attended by community nurses in the clients’ home. Barriers to delivery of this treatment include; client concordance and or suitability for bandaging including client habitus, (shape of legs), client lifestyle, clinician knowledge and clinicians physical ability to attend bandaging, in particular for obese clients with limited mobility who pose a manual handling risk to the clinician themselves. The use of IPC may assist in mitigating some of these concerns, therefore it would seem wise to explore the use of IPC within the home setting.

**Case presentation:**

This paper will present an original case report on the successful treatment of a woman living with chronic bilateral lower leg ulcers using IPC as an adjunct treatment in her home. This paper supports recommendations to explore the use of IPC therapy in the home setting, for treatment of chronic leg ulcers requiring compression.

**Conclusion:**

Use of IPC in the home is anticipated to improve client involvement, concordance, client outcomes and reduce risk to staff applying conventional compression bandaging systems, particularly for obese clients with limited mobility.

## Background

The incidence of Australians living with a chronic leg ulcer (CLU) has not been accurately determined. It is estimated that up to 3% of the Australian population will develop a chronic venous leg ulcer in their lifetime [1], with McGuiness [2] reporting that approximately 400,000 people in Australia will suffer a CLU at any one time. Prevalence of chronic leg ulceration is rising in Australian society as a result of our ageing population and increasing risk factors such as diabetes, obesity, peripheral vascular disease, congestive cardiac failure, arthritis, injury and respiratory diseases [3]. CLU is defined as a defect in the skin below the knee that does not progress through the normal phases of healing and generally is considered chronic when it persists for longer than three months [4]. Chronic wounds are a poorly recognised chronic disease that causes pain and suffering and costs the Australian healthcare system an estimated A$2.85 billion dollars per annum [5, 6]. The reported cost for acute management of CLUs alone was estimated at between A$553 and A$654 million in 2002 [7]. Not only does CLU pose a significant economic cost to the Australian healthcare system, but there is also a substantial personal cost [6]. Living with chronic wounds has a negative psychological impact on a person, often leading to loss of control, social isolation and depression [8]. To improve outcomes for individuals, they should be given the opportunity to understand their condition, be involved in their treatment, and have access to resources encouraging them to take responsibility for managing their wound where possible [9].

IPC is a device that uses an air pump to inflate and deflate an airtight garment placed around the legs, which mimics the rhythmic calf muscle contractures and promotes improved venous flow and lymphatic drainage [10]. IPC is also used as a treatment for deep vein thrombosis (DVT) prevention and it has been shown to improve healing of CLU [11, 12]. A review of the literature supported further exploration of the use of IPC as an adjunct therapy for treatment of CLU of venous aetiology [12, 13]. IPC should be utilised with a holistic approach to wound management encompassing client related factors, wound related factors, environmental factors, skills and knowledge of clinicians, resource and treatment related factors [14]. Concordance with traditional forms of compression bandaging and hosiery is a challenge faced by healthcare professionals in the provision of compression therapy [15, 16].

Although compression using bandages or hosiery and calf pump exercises is proven to help healing and prevent recurrence of ulcers, many people do not adhere to compression therapy [12, 17]. There are a variety of reasons for non-concordance including pain, discomfort and inadequate lifestyle advice by healthcare providers, as reported by clients with leg ulcers [18, 19], additional reasons outlined by Van Hecke et al. [19] for non-concordance are difficulties in applying compression, skin problems, uncomfortable footwear, cosmetic appearance of bandages and financial restrictions prompting AWMA to push for a subsidised scheme for compression therapy in Australia as the high cost of therapy puts it out of reach for many Australians [2].

Whether or not IPC is better than compression bandaging has not been established, but the consensus is IPC is better for healing ulcers than no compression and improves outcomes for people with CLU [12, 17]. This case report outlines one woman’s journey living with CLU and treatment using IPC in her home, which will be presented using the CARE checklist [20].

### Timeline


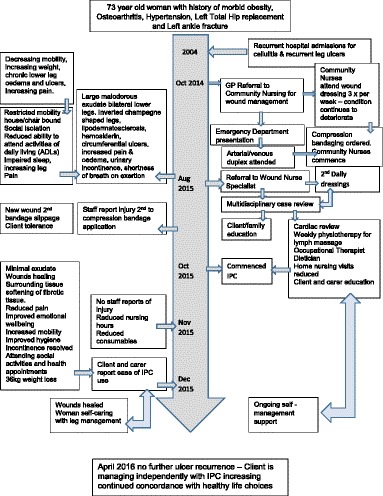


## Case presentation

### Patient information

An elderly woman was referred to Community Health in October 2014 for management of chronic lower leg ulcers (table 1). The woman had suffered recurrent hospital admissions throughout the prior ten years for lower leg cellulitis and leg ulcers, with three admissions within the previous two year period. A complex interplay of factors led to her ulcer development, including risk factors such as age and sex, relevant background history such as osteoarthritis, fractured left ankle, left total hip replacement, and morbid obesity leading to decreased mobility and calf pump action, causing chronic venous insufficiency of the superficial veins which also caused chronic lower leg oedema and lymphoedema. The woman reported the impact that the chronic ulcers were having on her life including; increasing pain, large amounts of odorous discharge, decreased mobility leading to increasing weight gain, lack of sleep, urinary incontinence, inability to shower, lack of independence, lack of energy, mood changes and social isolation.

Previous treatments had included antibiotic therapy and basic dressings with the General Practitioner (GP), hospital admissions for leg elevation and intravenous antibiotics. Access to specialised lymphoedema treatment was limited for two primary reasons. Firstly, she had become house bound with restricted transport to health specialists, and secondly, she lived in a geographically remote area. Community nurses commenced applying a two layer short stretch compression bandaging system in October 2014 as per previous hospital discharge instructions. The procedure required two community nurses to attend two to three times per week. A wound specialist review occurred in August 2015 following the woman’s discharge from the emergency department where she had presented for review for increasing pain, redness and swelling of her legs. It had been noted that she had not been receiving consistent compression bandaging. The community nurses reported adverse events from bandaging the woman’s legs including tissue damage from bandage slippage, friction and pressure and increased knee and thigh oedema with some nurses reporting personal injuries and difficulty with manual application of bandaging, the woman understood the need for compression but found it difficult to tolerate the bandaging due to pain at times.

### Physical exam

#### Diagnostic assessment

**Fig. 1:**
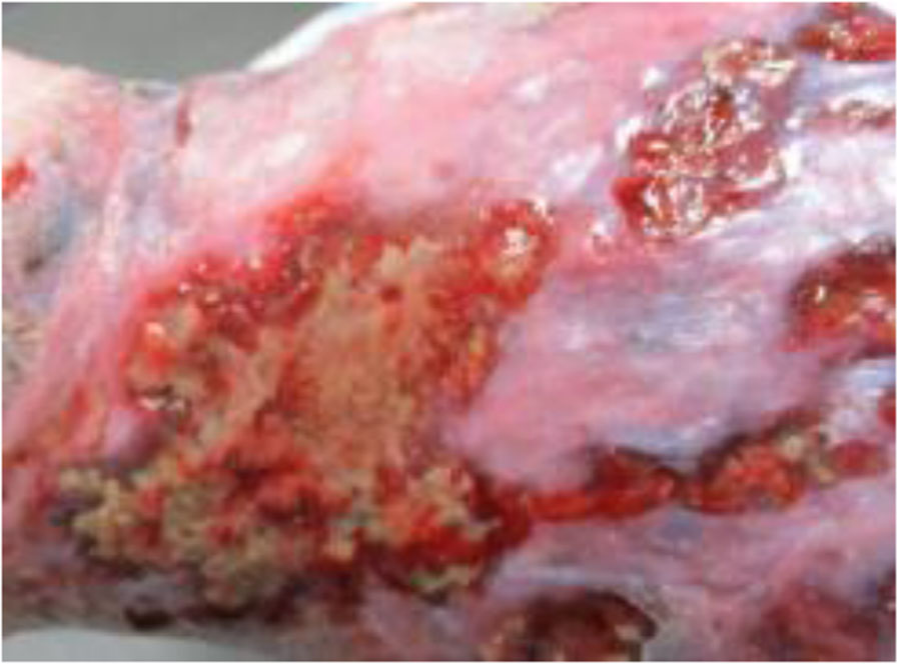
Tissue damage from bandage slippage posterior lower calf

**Fig. 2:**
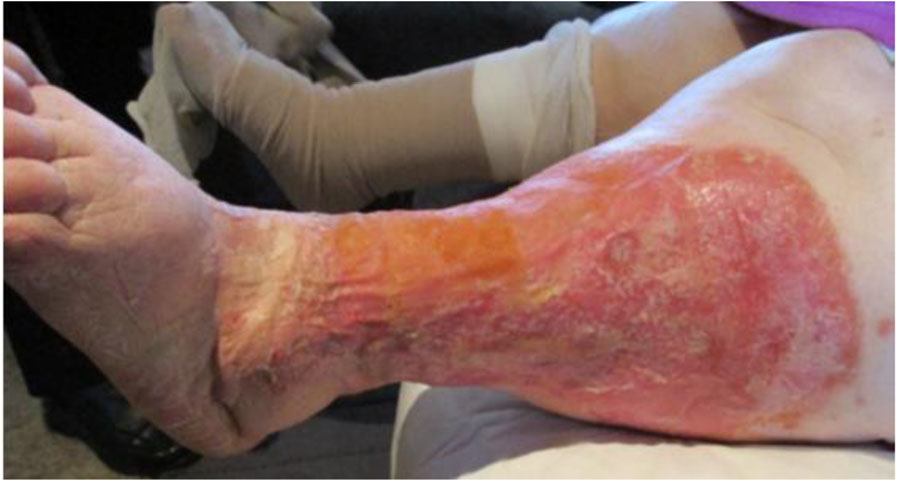
Pre IPC therapy: Circumferential ulceration and tissue maceration from continued oedema and large exudate leakage

The Wound Nurse Specialist examination in August 2015 noted significant oedema of the woman’s lower legs, thighs. Bilateral lower legs had circumferential superficial ulcers with a copious amount of malodorous greenish exudate on the removed dressing, the presence of pseudomonas was confirmed by a wound swab. The skin of both legs was thickened with fibrosis, lipodermatosclerosis, venous eczema, and hemosiderin staining with hyperkeratosis and surrounding tissue maceration (Figs. 1 & 2). The woman rated her wound and lower leg pain at an 8 on the Wound Assessment Tool pain scale (i.e. 0 equates to no pain to 10 extreme pain). Her right thigh circumference was 89cm and Left thigh 93cm and she had multiple skin folds with skin irritation because of poor hygiene and incontinence. She also had Papillomatosis wart like projections which were visualised on her inner thighs, her vital signs were stable and within acceptable limits whilst resting. Her height measured at 154cm with a weight of 149kg giving her a Body Mass Index (BMI) 62.83.

The woman’s mobility was limited requiring assistance to stand from the sitting position, she mobilised with a four wheeled walker for five steps with wide stiff legged gait she was unable to attend knee bends or heel lifts, client became short of breath on exertion. Because of her reduced mobility she had been unable to sleep in bed remaining predominantly chair bound. The woman scored 20 on the Waterlow pressure injury risk assessment, placing her at very high risk for pressure ulcer development with a score of 10 or above being at risk, 15 -19 high risk, 20 and above very high risk [21]. A newly developed ulcer was visualised on her posterior ankle to calf (Fig. 1.) most likely due to bandage slippage and the woman’s inability to lift her leg to remove the pressure. Her dorsalis pedis pulse felt weak but palpable, the arterial duplex results confirmed no occlusion in tri vessel arterial supply. Her nutrition was suboptimal and this consisted of a high carbohydrate, fat and sugar diet. The woman reported poor hydration due to her limited mobility, commenting that she avoided drinking because she couldn’t get to the toilet and was not having regular bowel motions.

#### Interventions

The intervention commenced following the Wound Nurse Specialist review in August of 2015 and multidisciplinary case review at the woman’s home. Involved in the review were the woman her carer’ the GP, Occupational Therapist, Physiotherapist, Dietician, Community Nurse and Wound Nurse Specialist where a management plan was developed promoting the woman’s involvement in her own wound care planning. The overall treatment was aimed at, reducing the oedema, preventing further pressure injuries, improving skin hygiene and nutritional support to achieve the woman’s goal of wound healing, improving mobility and increasing independence and social activity.

A bariatric hospital bed was arranged by the Occupational Therapist, enabling the woman to elevate her legs to promote venous return and reduce staff risk of injury during dressing changes. A shower chair was provided enabling the woman to attend showers to improve personal hygiene and her feelings of self-worth. Nutrition support was also provided to optimise her nutrition for wound healing whilst monitoring weight loss was guided by the dietician who worked with both the woman and her carer providing education and support for meal planning. The physiotherapist attended the woman’s home weekly to provide lymphatic drainage massage and exercise physiology including mobility training.

Medications were reviewed by a cardiologist who commenced her on oral furosemide 40mg twice per day. Safety clearance was provided by both a vascular surgeon and cardiologist to use IPC. IPC was commenced using bilateral thigh length leg garments at 40mmHg pressure, 2 cycles per minute, 40 -60 minute session duration attended three times per day. The woman was encouraged to increase frequency of sessions when she was able. The IPC therapy was attended at home by the woman and her carer’ following Initial session with the Wound Nurse Specialist to educate the woman and her carer’ on IPC use and pre-therapy breathing exercise. The woman continued leg elevation and calf pump exercises three times per day and commenced daily mobility with a four wheeled walker in conjunction with IPC.

Dressings were attended in the home second daily by community nurses for the first two weeks included in regime was leg hygiene, betadine wash, and PH neutral cream to surrounding tissue, Inadine (pharmaceutical topical antimicrobial) was applied to raw areas with an absorbent padding, tubi-fast to secure and tubi-grip. Home nursing visits were reduced to three times per week for weeks three to four, during this time community nurses continued to educate the woman and her carer to self - manage dressings. From week five to six frequency of home nursing visits were reduced to twice per week. Home nursing visits were reduced to weekly for the following six weeks. Fortnightly community nursing home visits to monitor and support self – management continued until the woman was discharged from community wound service in March of 2016.

#### Follow-up and outcome

**Fig. 3:**
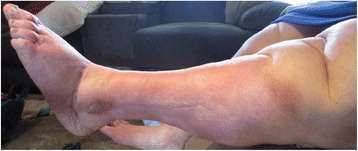
Week 6: post IPC therapy initiation; no exudate, complete epithelialisation, reduced oedema

**Fig. 4:**
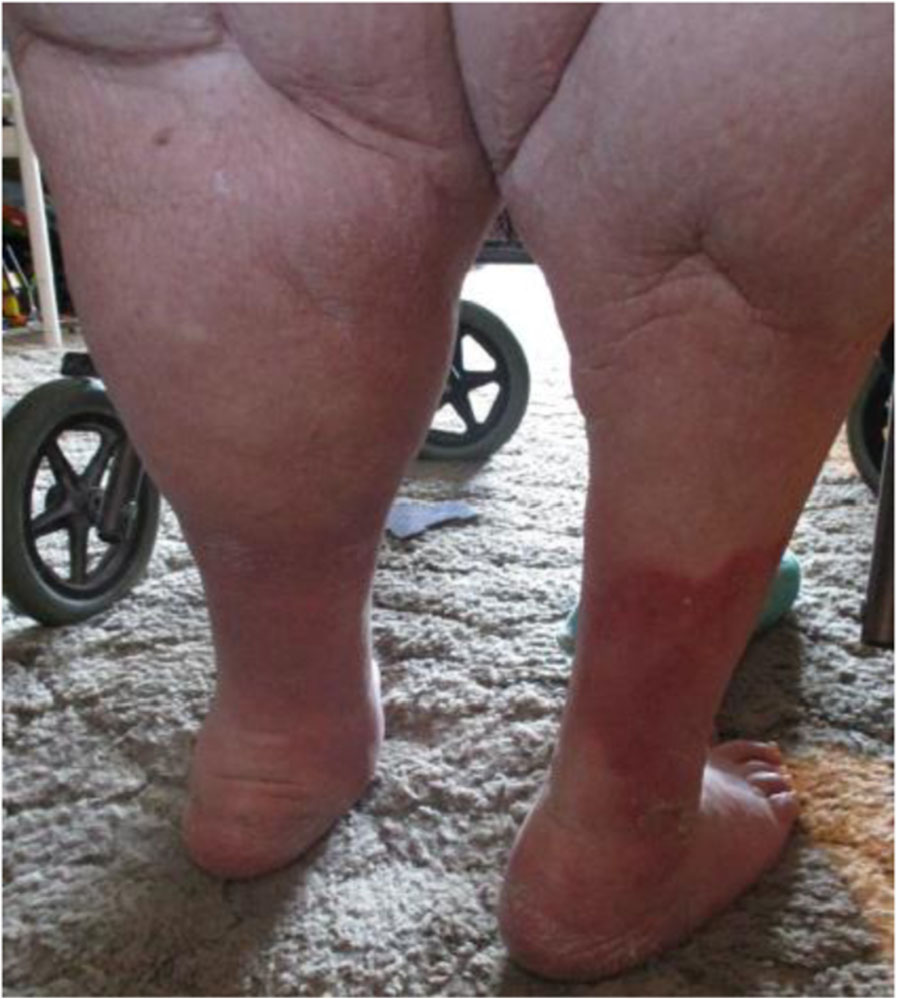
Week 10: client mobilising, reduced oedema fibrotic tissue softening

**Fig. 5:**
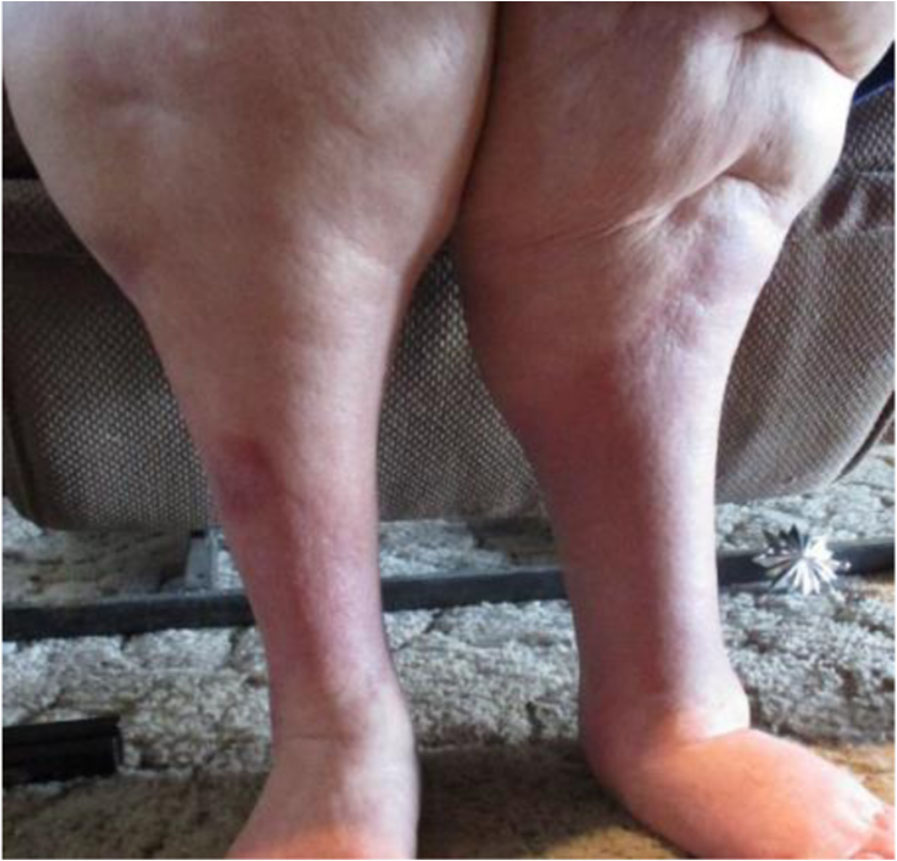
Week 12: no ulcerations or exudate leakage

Twelve weeks following the introduction of IPC therapy, this woman accomplished a significant weight loss of 36kg, lowering her BMI from 62.83 to 47.65. There was also a reduction of over 10cm in thigh circumference, her right thigh measured at 79cm and left thigh measured at 82cm. There was substantial wound healing achieved (complete epithelialisation with no exudate leakage or skin irritation) (Fig. 3) and wound pain scale score reduced to 0/10. There was noted improvement in skin condition and softening of sub-dermal tissue. The skin folds to her legs had reduced (Figs. 4 & 5) and this woman reported improved personal hygiene and self-esteem, she now enjoyed socialising outside the home.

Concordance with IPC therapy during this time was self - reported with the woman stating “only an occasional session was missed”. This woman was now able to bend her knees and attend heel lifts and continued daily mobilisation – walking around the outside of her house to the clothes line and back independently with a four wheeled walker. Her independence with activities of daily living was improved, the woman reported feeling proud of herself as she was managing to shower, dry and dress without assistance and reported being able to attend housework such as “cleaning out kitchen cupboards” which she hadn’t attended to in years her urinary incontinence resolved and bowel motions were regular.

This woman reported some initial discomfort with an increase in frequency of urination when first commenced on IPC, stating this settled within two weeks. Her Waterlow assessment was updated and scored 10, the reduced risk score predominantly resulting from improved mobility and continence. There were no reported episodes of infections during or after the twelve weeks IPC therapy. A follow up phone call was attended in April of 2016 where the woman confirmed her continued self-management with IPC and healthy life choices to prevent ulcer recurrence. The woman reported no further ulcers or infections had developed.

#### Patient perspective of using IPC at home twelve weeks on

When asked how she felt about her experience with her chronic wounds, before and after IPC she responded;

“I felt very depressed before because my legs kept leaking, they smelt bad and hurt a lot, I didn’t want to leave the house, soon I just wasn’t able to, and I couldn’t even walk to the toilet or wash myself. I found the IPC pump easy to use, at first my daughter had to put it on, now I can do it myself. My wounds are healed and I’m walking more every day, I can get to the toilet so don’t have accidents anymore. I have gone out with friends I haven’t seen for ages and can now play with my grandchildren, I feel much happier now”

## Discussion

It is widely accepted that CLU development is primarily attributed to CVI with Gold standard treatment including compression bandaging [13], in combination with other measures such as physiotherapy, diet, patient education, pressure management and antibiotics where required [1, 8, 21]. Developing treatment methods such as transcutaneous electrical nerve stimulation (TENS) has shown some improvement, with VLU healing in people with multiple co-morbidities [22] but further investigation of this method is required to improve level of evidence. TENS is not part of current Australian guideline recommendations (1) as such public access to this treatment is not available to clients of the service.

The practice of bandaging however is not always practical in clinical practice with a variety of barriers being identified as preventing effectiveness of static compression therapy (bandaging) [15, 19]. This case report outlined barriers this woman faced with traditional compression, the introduction of IPC enabled her access to treatment in the home. A good result was achieved in this individual case. The woman improved her quality of life, with complete resolution of wounds, improved mobility her pain resolved and independence increased. The costings of consumables and nursing hours in this instance were collected eight weeks pre and twelve weeks post IPC therapy showing a saving of approx. $250 per week for the community health service.

Because other treatment measures were used in combination with IPC in this instance and concordance was self-reported it is difficult to measure the significance IPC alone had on this woman’s wound healing outcomes. A review by Nelson et al. [12] suggests further trials are required to determine reliability of current evidence on IPC use for leg ulcer healing. This is the first documented study using IPC in the home for treatment of CLU. Results from this case, support the need to further explore the use of IPC as an adjunct therapy for leg ulcer management in the community setting. A full clinical trial of the use of IPC at home is in the planning stages.

## Abbreviations

CLU: Chronic leg ulcer
CVI: Chronic venous insufficiency
IPC: Intermittent pneumatic compression
VLU: Venous leg ulcer
